# Supplementary material for: Analysis of PPARγ Signaling Activity in Psoriasis
Source: Int J Mol Sci. 2021 Aug 10;22(16):8603. doi: 10.3390/ijms22168603 (PMC8395241; doi:10.3390/ijms22168603)
Supplement: Supplementary file 1 [file ijms-22-08603-s001.zip › Supplemental materials_Analysis of PPARg signaling activity in psoriasis/Pathway models/Models images and html files/Interleukin-17 and interleukin-22 signaling in psoriasis/2919.html]

CSF3


# Protein CSF3

|  |  |
| --- | --- |
| URN | urn:agi-llid:1440 |
| Total Entities | 0 |
| Connectivity | 4635 |
| Name | CSF3 |
| Description | colony stimulating factor 3 (granulocyte) |
| Notes | The protein encoded by this gene is a cytokine that controls the production, differentiation, and function of granulocytes. The active protein is found extracellularly. Alternatively spliced transcript variants have been described for this gene. [provided by RefSeq, May 2010] |
| Primary Cell Localization | Extracellular |
| Class | Ligand |

---

|  |  |
| --- | --- |
| Pathway | Neutrophil Recruitment and Priming |
|  | IL1B Expression Targets -> Nociception |
|  | Myeloid Progenitor Cell -> GEMM - CFU Surface Expression Markers |
|  | Proteins Involved in Atherosclerosis |
|  | Proteins Involved in Dilated Cardiomyopathy |
|  | Proteins Involved in Myocardial Ischemia |
|  | Proteins Involved in Myocarditis |
|  | Proteins Involved in Acne Vulgaris |
|  | IL17 Signaling in Psoriasis |
|  | Proteins Involved in Psoriasis |
|  | Neutrophil Recruitment in Sinovium in Gout |
|  | Proteins Involved in Osteoporosis |
|  | Proteins Involved in Noise-Induced Hearing Loss |
|  | Cytokines Trigger Otitis Media |
|  | Proteins Involved in Chronic Obstructive Pulmonary Disease |
|  | Proteins Involved in Pyelonephritis |
|  | Elevated Receptors -> Expression Targets in Bone and Joint |
|  | CSF3 Expression Targets |
|  | Clozapine Induced Granulocytopenia |
|  | F2 -> STAT1/NF-kB Expression Targets |
|  | Elevated Receptors -> Expression Targets in Appendix |
|  | Myeloid Derived Suppressor Cells in Cancer Immune Escape |
|  | Proteins with Altered Expression in Cancer Immune Escape |
|  | NOTCH -> Expression Targets in Skin |
|  | CD40 -> Expression Targets in Lymph Node |
|  | NOTCH -> Expression Targets in Thymus |
|  | Adenosine Expression Targets |
|  | ERK5/MAPK7 Signaling |
|  | TNF -> NF-kB Expression Targets |
|  | TLR1 -> 2/6 Expression Targets |
|  | IL1B Expression Targets |
|  | IL1A Expression Targets |
|  | pprarg neg, uknown targets, ps-positive |
|  | prarg neg,ukn expres targets, ps-positive |
|  | PPARG negative targets |
|  | pparg expr target\_regulators |
|  | Neighbors of disease exacerbation |
|  | Neighbors of tumor progression |
|  | Neighbors of cancer progression |
|  | Neighbors of inflammatory status |
|  | Neighbors of overall survival |
|  | Neighbors of disease severity |
|  | Neighbors of tumor size |
|  | Neighbors of lesion size |
|  | Neighbors of mortality |
|  | Neighbors of survival rate |
|  | Neighbors of infarct size |
|  | Neighbors of survival time |
|  | Neighbors of functional recovery |
|  | Neighbors of bacterial load |
|  | Neighbors of graft survival |
|  | Neighbors of therapeutic efficacy |
|  | Neighbors of lethality |
|  | Neighbors of tumor regression |
|  | Neighbors of recurrence-free survival |
|  | Neighbors of disease-free survival |
|  | Neighbors of tumor response |
|  | Neighbors of macrophage count |
|  | Neighbors of long-term survival |
|  | Neighbors of treatment outcome |
|  | Neighbors of bone mass |
|  | Neighbors of body weight |
|  | Neighbors of body weight gain |
|  | Neighbors of tumor recurrence |
|  | Neighbors of tumor promotion |
|  | Neighbors of neutrophil count |
|  | Neighbors of morbidity |
|  | Neighbors of mortality rate |
|  | Neighbors of exhaustion |
|  | Neighbors of bone mineral density |
|  | Neighbors of adverse outcome |
|  | Neighbors of lesion area |
|  | Neighbors of cell density |
|  | Neighbors of progression-free survival |
|  | Neighbors of early mortality |
|  | Neighbors of shear stress |
|  | Neighbors of viral load |
|  | Neighbors of leukocyte count |
|  | Neighbors of vascular density |
|  | Neighbors of muscle mass |
|  | Neighbors of cancer risk |
|  | Neighbors of systolic blood pressure |
|  | Neighbors of tumor resistance |
|  | Neighbors of blood glucose |
|  | Neighbors of ejection fraction |
|  | Neighbors of median survival |
|  | Neighbors of osteoclast number |
|  | Neighbors of T-cell count |
|  | Neighbors of energy expenditure |
|  | Neighbors of vascular tone |
|  | Neighbors of radiosensitivity |
|  | Neighbors of left ventricular ejection fraction |
|  | Neighbors of breast cancer risk |
|  | Neighbors of proliferation index |
|  | Neighbors of pregnancy outcome |
|  | Neighbors of functional status |
|  | Neighbors of neurological outcome |
|  | Neighbors of infarct area |
|  | Neighbors of recurrence rate |
|  | Neighbors of lymphocyte count |
|  | Neighbors of monocyte count |
|  | Neighbors of metabolic response |
|  | Neighbors of insulin level |
|  | Neighbors of bone volume |
|  | Neighbors of fractional shortening |
|  | Neighbors of disease exacerbation |
|  | Neighbors of tumor progression |
|  | Neighbors of cancer progression |
|  | Neighbors of inflammatory status |
|  | Neighbors of overall survival |
|  | Neighbors of disease severity |
|  | Neighbors of tumor size |
|  | Neighbors of lesion size |
|  | Neighbors of mortality |
|  | Neighbors of survival rate |
|  | Neighbors of infarct size |
|  | Neighbors of survival time |
|  | Neighbors of functional recovery |
|  | Neighbors of bacterial load |
|  | Neighbors of graft survival |
|  | Neighbors of therapeutic efficacy |
|  | Neighbors of lethality |
|  | Neighbors of tumor regression |
|  | Neighbors of recurrence-free survival |
|  | Neighbors of disease-free survival |
|  | Neighbors of tumor response |
|  | Neighbors of macrophage count |
|  | Neighbors of long-term survival |
|  | Neighbors of treatment outcome |
|  | Neighbors of bone mass |
|  | Neighbors of body weight |
|  | Neighbors of body weight gain |
|  | Neighbors of tumor recurrence |
|  | Neighbors of tumor promotion |
|  | Neighbors of neutrophil count |
|  | Neighbors of morbidity |
|  | Neighbors of mortality rate |
|  | Neighbors of exhaustion |
|  | Neighbors of bone mineral density |
|  | Neighbors of adverse outcome |
|  | Neighbors of lesion area |
|  | Neighbors of cell density |
|  | Neighbors of progression-free survival |
|  | Neighbors of early mortality |
|  | Neighbors of shear stress |
|  | Neighbors of viral load |
|  | Neighbors of leukocyte count |
|  | Neighbors of vascular density |
|  | Neighbors of muscle mass |
|  | Neighbors of cancer risk |
|  | Neighbors of systolic blood pressure |
|  | Neighbors of tumor resistance |
|  | Neighbors of blood glucose |
|  | Neighbors of ejection fraction |
|  | Neighbors of median survival |
|  | Neighbors of osteoclast number |
|  | Neighbors of T-cell count |
|  | Neighbors of energy expenditure |
|  | Neighbors of vascular tone |
|  | Neighbors of radiosensitivity |
|  | Neighbors of left ventricular ejection fraction |
|  | Neighbors of breast cancer risk |
|  | Neighbors of proliferation index |
|  | Neighbors of pregnancy outcome |
|  | Neighbors of functional status |
|  | Neighbors of neurological outcome |
|  | Neighbors of infarct area |
|  | Neighbors of recurrence rate |
|  | Neighbors of lymphocyte count |
|  | Neighbors of monocyte count |
|  | Neighbors of metabolic response |
|  | Neighbors of insulin level |
|  | Neighbors of bone volume |
|  | Neighbors of fractional shortening |
|  | Neighbors of cell development |
|  | Neighbors of immune response |
|  | Neighbors of cell population |
|  | Neighbors of T-cell development |
|  | Neighbors of inflammatory response |
|  | Neighbors of wound healing |
|  | Neighbors of angiogenesis |
|  | Neighbors of innate immune response |
|  | Neighbors of cell function |
|  | Neighbors of macrophage activation |
|  | Neighbors of cell formation |
|  | Neighbors of adaptive immune response |
|  | Neighbors of osteoclast development |
|  | Neighbors of epithelial to mesenchymal transition |
|  | Neighbors of immunity |
|  | Neighbors of cell proliferative response |
|  | Neighbors of chemotaxis |
|  | Neighbors of dendritic cell differentiation |
|  | Neighbors of cell survival |
|  | Neighbors of cell phenotype |
|  | Neighbors of T-cell proliferation |
|  | Neighbors of tissue remodeling |
|  | Neighbors of cell adhesion |
|  | Neighbors of T-cell activation |
|  | Neighbors of endothelial cell proliferation |
|  | Neighbors of fibrogenesis |
|  | Neighbors of tumor growth |
|  | Neighbors of dendritic cell development |
|  | Neighbors of tissue repair |
|  | Neighbors of cell count |
|  | Neighbors of cell infiltration |
|  | Neighbors of cell interaction |
|  | Neighbors of SMC proliferation |
|  | Neighbors of lumen formation |
|  | Neighbors of endothelial cell migration |
|  | Neighbors of adipogenesis |
|  | Neighbors of lipid storage |
|  | Neighbors of cell damage |
|  | Neighbors of pregnancy |
|  | Neighbors of monocyte differentiation |
|  | Neighbors of aging |
|  | Neighbors of cell motility |
|  | Neighbors of bone resorption |
|  | Neighbors of monocyte migration |
|  | Neighbors of fibroblast proliferation |
|  | Neighbors of adipocyte differentiation |
|  | Neighbors of microglial activation |
|  | Neighbors of neutrophil recruitment |
|  | Neighbors of regeneration |
|  | Neighbors of cell migration |
|  | Neighbors of cancer growth |
|  | Neighbors of stem cell proliferation |
|  | Neighbors of smooth muscle cell migration |
|  | Neighbors of hepatic regeneration |
|  | Neighbors of transendothelial migration |
|  | Neighbors of phagocytosis |
|  | Neighbors of immune system activation |
|  | Neighbors of leukocyte migration |
|  | Neighbors of leukocyte recruitment |
|  | Neighbors of osteoclast formation |
|  | Neighbors of monocyte recruitment |
|  | Neighbors of cell invasion |
|  | Neighbors of heart function |
|  | Neighbors of hemopoiesis |
|  | Neighbors of bone remodeling |
|  | Neighbors of ossification |
|  | Neighbors of T-cell function |
|  | Neighbors of immune system function |
|  | Neighbors of lung development |
|  | Neighbors of autophagy |
|  | Neighbors of monocyte adhesion |
|  | Neighbors of transcription activation |
|  | Neighbors of T-cell response |
|  | Neighbors of tumor immunity |
|  | Neighbors of hepatocyte proliferation |
|  | Neighbors of cell transdifferentiation |
|  | Neighbors of neuroprotection |
|  | Neighbors of stem cell differentiation |
|  | Neighbors of endothelial cell function |
|  | Neighbors of neuronal death |
|  | Neighbors of cell growth |
|  | Neighbors of ROS generation |
|  | Neighbors of epithelial cell proliferation |
|  | Neighbors of first trimester pregnancy |
|  | Neighbors of cancer cell growth |
|  | Neighbors of sensitization |
|  | Neighbors of hemato-encephalic barrier |
|  | Neighbors of macrophage function |
|  | Neighbors of cellular immune response |
|  | Neighbors of cellular senescence |
|  | Neighbors of colony formation |
|  | Neighbors of macrophage differentiation |
|  | Neighbors of cell differentiation |
|  | Neighbors of keratinocyte proliferation |
|  | Neighbors of stem cell migration |
|  | Neighbors of life span |
|  | Neighbors of psoriasis |
|  | Neighbors of inflammatory disease |
|  | Neighbors of rheumatoid arthritis |
|  | Neighbors of atherosclerosis |
|  | Neighbors of fibrosis |
|  | Neighbors of colitis |
|  | Neighbors of arthritis |
|  | Neighbors of inflammatory bowel disease |
|  | Neighbors of injury |
|  | Neighbors of atherogenesis |
|  | Neighbors of vascular remodeling |
|  | Neighbors of metastasis |
|  | Neighbors of carcinogenesis |
|  | Neighbors of multiple sclerosis |
|  | Neighbors of chronic inflammation |
|  | Neighbors of inflammation |
|  | Neighbors of tumor microenvironment |
|  | Neighbors of pathological angiogenesis |
|  | Neighbors of obesity |
|  | Neighbors of vascular disease |
|  | Neighbors of diabetes mellitus |
|  | Neighbors of asthma |
|  | Neighbors of pneumonia |
|  | Neighbors of hyperplasia |
|  | Neighbors of osteoarthritis |
|  | Neighbors of liver fibrosis |
|  | Neighbors of chronic obstructive pulmonary disease |
|  | Neighbors of myocardial infarction |
|  | Neighbors of leukocyte infiltration |
|  | Neighbors of reperfusion injury |
|  | Neighbors of airway inflammation |
|  | Neighbors of hepatitis |
|  | Neighbors of infection |
|  | Neighbors of vascular injury |
|  | Neighbors of renal injury |
|  | Neighbors of preeclampsia |
|  | Neighbors of macrophage infiltration |
|  | Neighbors of liver injury |
|  | Neighbors of neuroinflammation |
|  | Neighbors of sepsis |
|  | Neighbors of death |
|  | Neighbors of type 1 diabetes |
|  | Neighbors of encephalomyelitis |
|  | Neighbors of autoimmune disease |
|  | Neighbors of ischemic stroke |
|  | Neighbors of dermatitis |
|  | Neighbors of neutrophil infiltration |
|  | Neighbors of heart disease |
|  | Neighbors of diabetic nephropathy |
|  | Neighbors of lung injury |
|  | Neighbors of immunopathology |
|  | Neighbors of systemic sclerosis |
|  | Neighbors of hepatocellular carcinoma |
|  | Neighbors of autoimmunity |
|  | Neighbors of acute lung injury |
|  | Neighbors of gastric cancer |
|  | Neighbors of atherosclerotic plaque |
|  | Neighbors of experimental autoimmune encephalomyelitis |
|  | Neighbors of colorectal cancer |
|  | Neighbors of cardiac remodeling |
|  | Neighbors of breast cancer |
|  | Neighbors of ischemia |
|  | Neighbors of systemic lupus erythematosus |
|  | Neighbors of melanoma |
|  | Neighbors of collagen-induced arthritis |
|  | Neighbors of neoplasm invasion |
|  | Neighbors of heart failure |
|  | Neighbors of lung disease |
|  | Neighbors of pulmonary fibrosis |
|  | Neighbors of cardiovascular disease |
|  | Neighbors of stroke |
|  | Neighbors of cancer |
|  | Neighbors of cerebral ischemia |
|  | Neighbors of type 2 diabetes |
|  | Neighbors of neoplasm |
|  | Neighbors of endometriosis |
|  | Neighbors of neointima |
|  | Neighbors of vasculitis |
|  | Neighbors of atrophy |
|  | Neighbors of diet-induced obesity |
|  | Neighbors of myocardial fibrosis |
|  | Neighbors of synovitis |
|  | Neighbors of coronary artery disease |
|  | Neighbors of rupture |
|  | Neighbors of endothelial cell dysfunction |
|  | Neighbors of liver disease |
|  | Neighbors of prostate cancer |
|  | Neighbors of hypertrophy |
|  | Neighbors of osteolysis |
|  | Neighbors of ovarian cancer |
|  | Neighbors of pancreatic cancer |
|  | Neighbors of hypertension |
|  | Neighbors of intimal hyperplasia |
|  | Neighbors of allograft rejection |
|  | Neighbors of proteinuria |
|  | Neighbors of epithelial cell |
|  | Neighbors of keratinocyte |
|  | Neighbors of vascular smooth muscle cell |
|  | Neighbors of umbilical vein endothelial cell |
|  | Neighbors of monocyte |
|  | Neighbors of endometrial stromal cell |
|  | Neighbors of alveolar macrophage |
|  | Neighbors of synoviocyte |
|  | Neighbors of macrophage |
|  | Neighbors of adipocyte |
|  | Neighbors of activated monocyte |
|  | Neighbors of fibroblast |
|  | Neighbors of airway epithelial cell |
|  | Neighbors of PBMC |
|  | Neighbors of stromal cell |
|  | Neighbors of airway smooth muscle cell |
|  | Neighbors of osteoblast |
|  | Neighbors of pneumocyte |
|  | Neighbors of endometrium cell |
|  | Neighbors of microglia |
|  | Neighbors of trophoblast |
|  | Neighbors of intestine epithelium cell |
|  | Neighbors of mononuclear cell |
|  | Neighbors of hepatocyte |
|  | Neighbors of decidual cell |
|  | Neighbors of endothelial cell |
|  | Neighbors of dendritic cell |
|  | Neighbors of kidney tubule cell |
|  | Neighbors of bone marrow macrophage |
|  | Neighbors of peritoneal macrophage |
|  | Neighbors of neutrophil |
|  | Neighbors of smooth muscle myocyte |
|  | Neighbors of astrocyte |
|  | Neighbors of lung fibroblast |
|  | Neighbors of activated T-cell |
|  | Neighbors of mesothelial cell |
|  | Neighbors of mononuclear phagocyte |
|  | Neighbors of capillary endothelial cell |
|  | Neighbors of immunocompetent cell |
|  | Neighbors of granulocyte |
|  | Neighbors of cancer-associated fibroblast |
|  | Neighbors of myeloid cell |
|  | Neighbors of mesenchymal stroma cell |
|  | Neighbors of stem cell |
|  | Neighbors of tumor infiltrating macrophage |
|  | Neighbors of sinusoidal cell |
|  | Neighbors of B-cell |
|  | Neighbors of granulosa cell |
|  | Neighbors of leukocyte |
|  | Neighbors of T-cell |
|  | Neighbors of M1 macrophage |
|  | Neighbors of myofibroblast |
|  | Neighbors of mast cell |
|  | Neighbors of bone marrow stromal cell |
|  | Neighbors of spleen cell |
|  | Neighbors of hematopoietic cell |
|  | Neighbors of pericyte |
|  | Neighbors of phagocyte |
|  | Neighbors of lymphocyte |
|  | Neighbors of M2 macrophage |
|  | Neighbors of peritoneum cell |
|  | Neighbors of peripheral lymphocyte |
|  | Neighbors of activated B-cell |
|  | Neighbors of lung |
|  | Neighbors of liver |
|  | Neighbors of kidney |
|  | Neighbors of skin |
|  | Neighbors of intestine |
|  | Neighbors of lymph node |
|  | Neighbors of colon |
|  | Neighbors of heart |
|  | Neighbors of esophagus |
|  | Neighbors of brain |
|  | Neighbors of breast |
|  | Neighbors of stomach |
|  | Neighbors of islets of Langerhans |
|  | Neighbors of pancreas |
|  | Neighbors of lymphatic system |
|  | Neighbors of decidua |
|  | Neighbors of peritoneum |
|  | Neighbors of allantois |
|  | Neighbors of uterine cervix |
|  | Neighbors of arteriole |
|  | Neighbors of nose |
|  | Neighbors of mammary gland |
|  | Neighbors of bladder |
|  | Neighbors of nervous system |
|  | Neighbors of mediastinal lymph node group |
|  | Neighbors of NF-kB family |
|  | Neighbors of IL1 family |
|  | Neighbors of PPAR |
|  | Neighbors of Jun/Fos |
|  | Neighbors of NFKBI |
|  | Neighbors of PKC |
|  | Neighbors of PI3K |
|  | Neighbors of mitogen-activated protein kinase |
|  | Neighbors of IFNAR ligand |
|  | Neighbors of TLR |
|  | Neighbors of cytokine |
|  | Neighbors of interferon |
|  | Neighbors of interleukin |
|  | Neighbors of MEK1/2 |
|  | Neighbors of growth factor |
|  | Neighbors of ovalbumin |
|  | Neighbors of LDL |
|  | Neighbors of protein tyrosine kinase |
|  | Neighbors of LXR |
|  | Neighbors of inflammatory cytokine |
|  | Neighbors of IL23 |
|  | Neighbors of histone deacetylase |
|  | Neighbors of IL1R |
|  | Neighbors of Notch |
|  | Neighbors of prostaglandin-endoperoxide synthase |
|  | Neighbors of immunoglobulin |
|  | Neighbors of CD3 complex |
|  | Neighbors of TORC1 |
|  | Neighbors of histone acetyltransferase |
|  | New Pathway (4) |
|  | 2\_Interleukin-17 and interleukin-22 signaling in psoriasis |
|  | drug-target moltransport |
|  | Interleukin-17 and interleukin-22 signaling in psoriasis |

---

|  |  |
| --- | --- |
| MedScan ID | 1440 |

---

|  |  |
| --- | --- |
| LocusLink ID | 1440 |
|  | 12985 |
|  | 25610 |

---

|  |  |
| --- | --- |
| Alias | granulocyte growth factor |
|  | colony stimulation factor 3 isoform b |
|  | CSF3OS |
|  | colony stimulation factor III |
|  | MGC45931 |
|  | granulocyte colony-simulation factor |
|  | granulocyte growth factors |
|  | colony stimulating factor 3 isoform c |
|  | MGI-IG |
|  | G-CSF receptor ligand |
|  | Granulocyte colony-stimulating factor |
|  | colony stimulating factor 3 isoform a |
|  | G-CSF |
|  | colony stimulating factor,granulocyte |
|  | Csfg |
|  | colony stimulating factor III |
|  | OTTHUMP00000164327 |
|  | CSF3\_MOUSE |
|  | GCSF |
|  | colony stimulating factor 3 isoform b |
|  | chromosome 17 open reading frame 33 |
|  | OTTMUSP00000006785 |
|  | RP23-395E10.4 |
|  | Pluripoietin |
|  | colony stimulating factor 3 |
|  | colony stimulation factor 3 isoform a |
|  | Granulocyte colony-stimulation factor |
|  | colony stimulation factor 3 isoform c |
|  | CSF3 |
|  | colony stimulation factor 3 (granulocyte) |
|  | C17orf33 |
|  | granulocyte colony-stimulating factor receptor ligand |
|  | granulocyte colony-simulating factor |
|  | colony stimulation factor 3 |
|  | colony stimulating factor 3 (granulocyte) |
|  | filgrastim |
|  | granulocyte-colony stimulating factor |
|  | lenograstim |

---

|  |  |
| --- | --- |
| GO ID | 0005125 |
|  | 0019899 |
|  | 0005130 |
|  | 0008083 |
|  | 0071345 |
|  | 0071222 |
|  | 0019221 |
|  | 0030851 |
|  | 0006955 |
|  | 0007275 |
|  | 1901215 |
|  | 0051091 |
|  | 2000251 |
|  | 0030838 |
|  | 0008284 |
|  | 0045639 |
|  | 0033138 |
|  | 0050731 |
|  | 0014068 |
|  | 0032092 |
|  | 0051897 |
|  | 0045944 |
|  | 0045471 |
|  | 0005576 |
|  | 0005615 |
|  | 0010469 |
|  | 0042993 |

---

|  |  |
| --- | --- |
| KEGG ID | hsa:1440 |
|  | mmu:12985 |
|  | rno:25610 |

---

|  |  |
| --- | --- |
| Organism | Homo sapiens {Organism urn:agi-taxid:9606} |
|  | Mus musculus {Organism urn:agi-taxid:10090} |
|  | Rattus norvegicus {Organism urn:agi-taxid:10116} |
|  | Homo sapiens |
|  | Mus musculus |
|  | Rattus norvegicus |

---

|  |  |
| --- | --- |
| Mouse chromosome position | 11 62.45 cM |

---

|  |  |
| --- | --- |
| OMIM ID | 138970 |

---

|  |  |
| --- | --- |
| Rat chromosome position | 10q31 |

---

|  |  |
| --- | --- |
| Hugo ID | 2438 |
|  | HGNC:2438 |

---

|  |  |
| --- | --- |
| Human chromosome position | 17q21.1 |
|  | 17q11.2-q12 |

---

|  |  |
| --- | --- |
| Swiss-Prot Accession | P09919 |
|  | Q8N4W3 |
|  | Q6FH65 |
|  | P09919.1 |
|  | P09920 |
|  | Q0VB73 |
|  | P09920.1 |
|  | A8MXR7 |
|  | P97712 |

---

|  |  |
| --- | --- |
| PIR ID | A24573 |
|  | A25093 |
|  | A29536 |

---

|  |  |
| --- | --- |
| GenBank ID | NC\_000017 |
|  | NR\_033662 |
|  | NM\_000759 |
|  | NP\_000750 |
|  | NM\_172220 |
|  | NP\_757374 |
|  | NM\_172219 |
|  | NP\_757373 |
|  | NM\_001178147 |
|  | NP\_001171618 |
|  | AC090844 |
|  | AF388025 |
|  | AAK62469 |
|  | CH471152 |
|  | EAW60626 |
|  | EAW60627 |
|  | EAW60628 |
|  | EU660495 |
|  | ACF41164 |
|  | FB704344 |
|  | CAS03439 |
|  | FJ854694 |
|  | ACP21316 |
|  | M13008 |
|  | AAA03056 |
|  | X03656 |
|  | CAA27291 |
|  | AB102881 |
|  | BAD89410 |
|  | AK290247 |
|  | BAF82936 |
|  | AK298116 |
|  | BAG60399 |
|  | AK313509 |
|  | BAG36289 |
|  | BC033245 |
|  | AAH33245 |
|  | CB127794 |
|  | CD013926 |
|  | CR541891 |
|  | CAG46689 |
|  | HM132049 |
|  | ADI49832 |
|  | KP271008 |
|  | AJC19277 |
|  | M17706 |
|  | AAA35882 |
|  | M20922 |
|  | AAA66353 |
|  | X03438 |
|  | CAA27168 |
|  | X03655 |
|  | CAA27290 |
|  | P09919 |
|  | NC\_000077 |
|  | XM\_006532128 |
|  | XP\_006532191 |
|  | NM\_009971 |
|  | NP\_034101 |
|  | AL590963 |
|  | CH466556 |
|  | EDL16160 |
|  | X05402 |
|  | CAA28986 |
|  | AK145177 |
|  | BAE26276 |
|  | BC120759 |
|  | AAI20760 |
|  | BC120761 |
|  | AAI20762 |
|  | M13926 |
|  | AAA37672 |
|  | P09920 |
|  | NC\_005109 |
|  | NM\_017104 |
|  | NP\_058800 |
|  | AC\_000078 |
|  | AAHX01065923 |
|  | AC119462 |
|  | CH473948 |
|  | EDM05943 |
|  | U37101 |
|  | AAC52915 |
|  | NC\_018928 |
|  | AMYH02033391 |
|  | AC\_000033 |
|  | AAHY01095698 |
|  | XM\_006247265 |
|  | XP\_006247327 |
|  | AC\_000149 |
|  | ABBA01031851 |
|  | AABR06065504 |

---

|  |  |
| --- | --- |
| Swiss-Prot ID | CSF3\_HUMAN |
|  | CSF3\_MOUSE |

---

|  |  |
| --- | --- |
| Cell Localization | Secreted |
|  | Extracellular |

---

|  |  |
| --- | --- |
| Ensembl ID | ENSG00000108342 |
|  | ENSP00000225474.2 |
|  | ENST00000225474.6 |
|  | ENSP00000464421.1 |
|  | ENST00000583218.5 |
|  | ENSP00000377705.3 |
|  | ENST00000394149.7 |
|  | ENSP00000377704.3 |
|  | ENST00000394148.7 |
|  | ENST00000331769.6 |
|  | ENSMUSG00000038067 |
|  | ENSMUSP00000037762.2 |
|  | ENSMUST00000038886.2 |
|  | ENSRNOG00000008525 |
|  | ENSRNOP00000011509.3 |
|  | ENSRNOT00000011509.3 |
|  | ENSP00000225474 |
|  | ENST00000225474 |
|  | ENSP00000464421 |
|  | ENST00000583218 |
|  | ENSP00000377705 |
|  | ENST00000394149 |
|  | ENSP00000377704 |
|  | ENST00000394148 |
|  | ENST00000331769 |
|  | ENSMUSP00000037762 |
|  | ENSMUST00000038886 |
|  | ENSRNOP00000011509 |
|  | ENSRNOT00000011509 |

---

|  |  |
| --- | --- |
| MGI ID | MGI:1339751 |
|  | 1339751 |

---

|  |  |
| --- | --- |
| RGD ID | 2426 |

---

|  |  |
| --- | --- |
| Unigene ID | Hs.2233 |
|  | Mm.1238 |
|  | Rn.53973 |

---

|  |  |
| --- | --- |
| Homologene ID | 7677 |

---

|  |  |
| --- | --- |
| Shape | Rhomb |

---
